# Report on fraying resilience among the Ontario Registered Practical Nurse Workforce in long‐term care homes during COVID‐19

**DOI:** 10.1002/nop2.1678

**Published:** 2023-02-25

**Authors:** Denise M. Connelly, Nancy Snobelen, Anna Garnett, Nicole Guitar, Cecilia Flores‐Sandoval, Samir Sinha, Jen Calver, Diana Pearson, Tracy Smith‐Carrier

**Affiliations:** ^1^ School of Physical Therapy Western University London Ontario Canada; ^2^ WeRPN Mississauga Ontario Canada; ^3^ Arthur Labatt Family School of Nursing Western University London Ontario Canada; ^4^ Health and Rehabilitation Sciences Western University London Ontario Canada; ^5^ University of Toronto Toronto Ontario Canada; ^6^ Lambton College Sarnia Ontario Canada; ^7^ School of Humanitarian Studies Royal Roads University, Canada Research Chair (Tier 2) Victoria British Columbia Canada

**Keywords:** COVID‐19, engagement and resilience, geriatrics, long‐term care, practical nursing

## Abstract

**Aim:**

Registered Practical Nurses (RPNs) are frontline healthcare providers in Ontario long‐term care (LTC) homes. Throughout COVID‐19, RPNs working in LTC homes experienced prolonged lockdowns, challenging working conditions, and inadequate resource allocation. This study aimed to describe the personal and professional resilience of RPNs working in LTC during the COVID‐19 pandemic.

**Design:**

An open cross‐sectional online survey containing the Connor–Davidson Resilience Scale, Resilience at Work Scale®, and Resilience at Work Team Scale®.

**Methods:**

The survey was distributed by the RPN Association of Ontario (WeRPN) to approximately 5000 registered members working in Ontario LTC homes.

**Results:**

A total of 434 respondents participated in the survey (completion rate = 88.0%). Study respondents scored low on measures of resilience and reported extreme levels of job (54.5%) and personal (37.8%) stress. Resources to support self‐care and work‐life balance, build capacity for team‐based care practice(s) are needed.

## 1 INTRODUCTION

Registered Practical Nurses (RPNs) have been at the forefront of the Coronavirus Disease 2019 (COVID‐19) pandemic as the largest regulated health professional workforce in Ontario long‐term care (LTC) homes (Odom‐Forren, 2020). The LTC sector has been significantly impacted by the ongoing COVID‐19 pandemic due in part to insufficient processes for pandemic preparedness and the historical challenges, such as chronic staffing shortages, low staffing levels, heavy workloads, punitive measures for staff who are sick, structural deficiencies, and lack of infection control processes (Marrocco et al., 2021; McGilton et al., 2020). Awareness of chronic under staffing and funding of the LTC sector came to the fore with the Romanow Report (Romanow, 2000). Two decades later, the demand for LTC nurses and services is outpacing the investment and organization in infrastructure, and policy (Bell, 2021). Further, the high rate of turnover in nurses, in a profession that relies heavily on skill, knowledge and experience acquisition, is a major source of inefficiency (David & Brachet, 2011). Relative to other health sectors, the COVID‐19 pandemic disproportionately impacted Canadian LTC homes, residents, families, and all staff working in them, including unregulated staff (Hsu et al., 2020). A total of 72% of all COVID‐19‐related deaths in Ontario occurred in LTC homes while only 54% of the health care providers working in LTC agreed that COVID‐19 recommendations were a feasible strategy for managing the pandemic (Siu et al., 2020). As a result, the public became starkly aware of the social inequities in the LTC sector (Siu et al., 2020) and the indispensable nature of the approximately 5000 RPNs (Lankshear & Rush, 2018) providing nursing care to older adults living in Ontario LTC homes. The pandemic added significant strain to RPNs working in LTC who historically had experienced high levels of burnout, turnover and working in an environment that is inadequately staffed (White et al., 2021).

## 2 BACKGROUND

With ever‐rising reports of stress and burnout in the nursing profession the concept of resilience has emerged as an essential attribute for nurses' wellbeing, gaining attention in both research and clinical practice (Cooper et al., 2020). Resilience is key for health professionals to allow them to successfully, and continuously, navigate complex and stressful work environments (Huey & Palaganas, 2020). Low resilience in the nursing workforce has been found to cause increased health costs, low staff retention and poorer patient outcomes (Mealer et al., 2014; Potter et al., 2013; Rushton et al., 2015). Resilience is not exclusively an individual trait and is largely impacted by the quality of a person's social and physical ecology (Ungar, 2011). For the purposes of conceptualizing resilience in this research, we used the ecological model of resiliency proposed by Ungar (2018), in which resilience is understood to be a “sequence of systemic interdependent interactions through which actors (whether persons, organisms, or ecosystems) secure the resources required for sustainability in stressed environments” (p. 2). Personal resilience is conceptualized as “a process by which people ‘bounce back’ from adversity, frustration and misfortune using the psychological and biological strengths humans employ to cope with challenges and threats” (Newman, 2003, p. 42). Similarly, professional resilience addresses the capacity of individuals to thrive in demanding workplace situations, exemplified by attitude and willingness to act in responding to difficult situations (Be You, 2020).

### 2.1 Long‐term care

LTC presents a stressful work environment with increasing medical complexities, structural deficiencies and resources, and insufficient staffing levels (Siu et al., 2020). According to the Long‐Term Care Staffing Study (2020), the healthcare sector ranks second highest for injuries resulting in time lost in Ontario, and people working in LTC are among the most at risk for physical injury in the healthcare sector (Ministry of Long‐Term Care, 2020). As of October 2020, nearly three quarters of Canada's COVID‐19‐related deaths had occurred in LTC (Siu et al., 2020). Evidence suggests that mortality risk in older adults in Ontario is concentrated in LTC, and this risk has increased sharply over the course of the pandemic (Fisman et al., 2020). Researchers from the COVID‐19 Ontario Remodelling Group advise that early identification of risk requires a focus on testing and provision of personal protective equipment to staff in LTC and restructuring the LTC workforce to prevent the spread of COVID‐19 (Fisman et al., 2020). Ontario rates of mortality in LTC are greater than that in other provinces, such as British Columbia, where researchers have suggested there was greater preparedness compared with Ontario: there was better coordination between LTC, public health, and hospitals; greater funding of LTC; more care hours for residents; fewer shared rooms; more non‐profit facility ownership; and more comprehensive inspections (Liu et al., 2020).

### 2.2 Resilience in nurses

Jackson et al. (2007) views resilience as a quality that is necessary to succeed in nursing and is “favourable to build… as a strategy for assisting nurses to survive and thrive” (p. 7). A review of healthcare worker resilience during the COVID‐19 pandemic (Baskin & Bartlett, 2021) suggests that building resilience in nurses and other healthcare workers can serve as a protective factor against negative outcomes related to the job, including burnout, anxiety, and depression, and can improve patient outcomes. The integrative review examined 191 studies that assessed resilience during COVID‐19. Results demonstrated that resilience scores of nurses in some countries (i.e., The United States; Petzel, 2021) suggest a decrease in nurse resilience, and nurses in other countries (i.e., China; Lyu et al., 2020) suggest an increase, when compared with pre‐pandemic levels.

Further, evidence from a cross‐sectional study of 185 frontline nurses during the COVID‐19 pandemic suggests a relationship between frontline nurses' psychosocial status, satisfaction with life and resilience (Zakeri et al., 2021). In this study, nurses worked in intensive care units, the general ward, or other related medical departments in Iran. Non‐resilience, as measured by a mean score of 59.87 on the CD‐RISC, was significantly associated with higher rates of psychological disorders. These findings implicate resilience as a factor related to nurses' mental health and suggest that it should be considered when supporting nurses during a crisis such as COVID‐19.

### 2.3 The current study

Conceptualizing resilience in RPNs as influenced by individual, professional, and workplace factors is useful in assessing the professional and personal social, emotional, psychological, physical, and organizational/workplace effects of the COVID‐19 pandemic. Therefore, the purpose of this study was to explore how RPNs in LTC were managing stress, working conditions, and building self‐care networks to identify the components of personal, professional, and organizational resilience in times of the COVID‐19 pandemic. RPNs in Ontario earn a diploma in Practical Nursing by taking a program of four semesters over two years in a college program leading to a diploma in Practical Nursing (RNAO, 2022). The COVID‐19 pandemic presents a unique opportunity to study our current gap in knowledge about the resilience of nurses working in LTC. Understanding the existing state of resilience for RPNs in LTC homes and identifying areas most challenging for RPNs will support the development of practice resources, recommendations for practice guidelines, inform institutional and governmental action plans, and influence policy change. Identifying and developing supports for unmet needs in sustaining resilience is critical to maintaining and engaging this workforce in LTC (Clark et al., 2020).

## 3 METHOD

### 3.1 Survey design and development

Qualtrics XM (Provo, UT) software was used to conduct an open online survey. The survey could be accessed and completed using a computer or smartphone and was accessible between April 13 and August 31, 2021. No translations from English were distributed. No incentives were provided for completion of the survey. Responses were securely stored on a firewall protected computer.

Survey items included multiple choice, Likert scales, and Yes/No questions, with some instances of optional open text boxes for written responses. The cross‐sectional survey collected demographic information about participants' age, gender, living situation, marital status, race and/or ethnicity, years of experience as an RPN, employment status, job title, personal COVID‐19 infection status, rate of COVID‐19 occurrence in their workplace(s), potential changes to living situation and household income, and work location, duties, and responsibilities during the pandemic. Respondents were also asked to rate their current physical and mental health status when compared with before the pandemic, and the levels of job and personal stress they experienced since January 2020. The survey was seven pages in total and the number of questions per page ranged from 1 to 42. A total of 121 questions were presented to respondents. Respondents were given the option to navigate backwards in the survey, to skip questions, or not give a response to a question. Adaptive questioning was not used. No time cut‐off for the completion of the survey was allocated.

### 3.2 Resilience measures

As a component of the online survey, respondents were asked to complete three resilience scales to assess their personal resilience, personal resilience at work and team‐based professional resilience at work; specifically using the Connor–Davidson Resilience Scale (CD‐RISC‐25; Connor & Davidson, 2003), Resilience at Work Scale® (R@W; Winwood et al., 2013), and the Resilience at Work Team Scale® (TR@W; McEwen & Boyd, 2018), respectively.

The CD‐RISC‐25 can distinguish resilient people from non‐resilient people in clinical and non‐clinical groups and can be used in research and clinical situations (Connor & Davidson, 2003). The CD‐RISC‐25 measures “personal competence, trust in one's instinct and tolerance of negative affect, positive acceptance of change and safe relationships, control, and spiritual influences” (Manzano & Ayala, p. 246). The scale contains 25 items rated on a 4‐point Likert scale ranging from “not true at all” (0) to “true nearly all the time” (4). Total CD‐RISC‐25 score ranges from 0 to 100, with higher scores indicating greater personal resilience and a cut‐off ≥80 is used to characterize the presence of personal resilience (Connor & Davidson, 2003). In their original research, 80 was established as a cut‐off score from a sample that contained a “community sample, primary care outpatients, general psychiatric outpatients, clinical trial of generalized anxiety disorder, and two clinical trials of PTSD” (Connor & Davidson, 2003, p. 1). More recent work has established a mean score of 73% on the CD‐RISC‐25 for nurses working in intensive care units in New Zealand (Yu et al., 2019), 71% for nurses working in Iran responding to the COVID‐19 pandemic (Afshari et al., 2021), and 52% in an American sample of nurses working in LTC (Lin et al., 2021). Connor and Davidson (2003) have reported the Cronbach's alpha of the CD‐RISC‐25 scale to be 0.89, with a reliability coefficient of 0.87 reported for this scale through test–retest reliability in a four‐week interval (Connor & Davidson, 2003). The scale has been deemed to have sound validity and reliability (Cronbach's alpha = 0.89; Derakhshanrad et al., 2014).

In previous research from New Zealand of nurses working in intensive care units (*N* = 93), a CD‐RISC‐25 mean score of 73% was found (SD = 9.6; Yu et al., 2019). The study sample mean age was 33.9 ± 9.6 years old, with 72.0% of the sample between 20–34 years of age. A total of 73% of the sample reported being female. Similarly, data from a sample of hospital nurses (*N* = 321) in Iran responding to the COVID‐19 pandemic demonstrated a group mean score of 71% (SD = 14.1; Afshari et al., 2021). Approximately 60% of this sample was female and the 20–30‐year age group was the largest comprising 54% of their sample. In contrast, a sample of American nurses working in LTC and rehabilitation settings (*N* = 120) demonstrated a group mean score of 52% (SD = 10.42; Lin et al., 2021). A total of 85% of their participants were female, with a mean age of 42.69 years and an unreported standard deviation.

Moreover, the Resilience at Work Scale® (R@W scale; Winwood et al., 2013) was used to measure the sample's personal resilience in the workplace. The R@W scale is a reliable 20‐item tool that measures seven domains of resilience in the context of work (i.e., Living Authentically, Finding Your Calling, Maintaining Perspective, Managing Stress, Interacting Cooperatively, Staying Healthy, and Building Networks; McEwen, 2019a, 2019b). Each item is rated on a seven‐point Likert scale ranging from “strongly disagree” (0) to “strongly agree” (6) with two items reverse‐scored. Higher total and subscale scores are indicative of higher resilience (possible range from 0–120; Winwood et al., 2013). On the R@W scale, previous research shows a mean standardized score of 70.27 (*N* = 482, SD = 11.53) among mental health nurses (Delgado et al., 2020). In this sample, the Living Authentically subscale (i.e., maintaining personal values, use personal strengths, and have good emotional awareness and regulation at work) had the highest mean score at 79.12 (SD = 12.30), and the Maintaining Perspective subscale (i.e., having the capacity to reframe setbacks, maintain a solution‐focus, and manage negativity) had the lowest mean score (*M* = 52.44, SD = 16.93). Similarly, in a different study, across six hospitals in the western United States (*N* = 48, mean age = 48) a mean score of 4.2 on the 7‐point Likert scale on the R@W has been reported (Carpio et al., 2018). The highest scoring subscale was also Living Authentically with a score of 5.3 (SD = 0.4), whereas the lowest was Maintaining Perspective, with a score of 3.1 (SD = 1.0). Therefore, multiple studies have previously shown that the capacity to focus on solutions at work, reframe difficulties and/or manage negative thinking achieve lower scores than domains capturing individuals' capacity for emotional awareness and self‐regulation.

The Resilience at Work Team Scale® (TR@W) scale is a 42‐item scale based on team‐based professional behaviours that promote individual behaviours encompassed in the R@W scale (i.e., elements of work engagement, emotional exhaustion, and team performance; McEwen & Boyd, 2018). The team‐based professional resilience scale includes seven subscales (i.e., Resourceful, Robust, Perseverance, Self‐Care, Capability, Connected, and Alignment). The team scale compliments and builds on the individual scale, facilitating a more comprehensive assessment of the individual at work. Less data are available in the literature for comparative purposes on the TR@W scale; however, McEwen and Boyd (2018) reported an average score of 4.49/7 (SD = 1.21; *N* = 344, mean age = 45, 80% female) on the 7‐point Likert scale on the TR@W for participants representing three industry sectors (i.e., state government, private, and not‐for‐profit).

### 3.3 Sample and Recruitment

RPNs working, or who had worked, in LTC homes in Ontario since January 2020 during the COVID‐19 pandemic were eligible and invited to participate in the study. Nursing students and other categories of nurses (e.g., Registered Nurses) were not eligible. Respondents were recruited through their professional association, the Registered Practical Nurses Association of Ontario (WeRPN). WeRPN sent a series of email invitations, that included the online survey link, over a 5‐month period, to approximately 5000 potential respondents currently catalogued as working in LTC homes in Ontario (Lankshear & Rush, 2018). Postings for the online survey were also advertised through the WeRPN newsletter, and social media channels (e.g., Facebook, Instagram, Twitter, and LinkedIn). A reminder email, sent by the WeRPN, was sent 2‐weeks after the initial email to encourage participation as recommended by Sammut et al. (2021). No direct contact was made with potential respondents and survey responses were anonymous. The collection of additional system data (e.g., respondent's IP address, cookies and location) was disabled using Qualtrics software, which uses encryption technology and restricted access authorizations to protect all data collected. No other log file analyses were used. The use of non‐probabilistic sampling, due to the physical and fiscal constraints of obtaining province‐wide access to individual contact information, prevented the calculation of a participation rate (i.e., we are unable to determine how many eligible people were exposed to our invitation to participate; Couper, 2000; AAPOR, 2010). Informed consent to participate was obtained on the landing page of the online survey.

### 3.4 Data management and statistical analyses

Survey data were exported from Qualtrics and organized in Excel software. Data analyses were completed using SPSS Version 25 (IBM). It was determined a priori that only questionnaires that were ≥80% complete would be analysed. Descriptive statistics were used to analyse responses. Any missing data from responses that were between 80% and 100% complete was excluded in descriptive statistic calculations. In the absence of normative data for the R@W and TR@W scales, comparison with other data found with nursing populations will be used for comparison.

### 3.5 Research reporting checklist

The Checklist for Reporting Results of Internet E‐Surveys (CHERRIES) was used in the writing of this manuscript (Eysenbach, 2004; see Appendix 1).

## 4 RESULTS

A total of 434 RPNs consented to participate in the survey; 51 surveys were <80% complete and were therefore excluded from data analysis. Additionally, one respondent was removed who indicated they were not an RPN. Accordingly, the total number of respondents who consented to participating in the survey was *N* = 381 (completion rate of survey = 88.0%; see Table 1 for participant demographic characteristics). Note that a view rate and a participation rate were not applicable. Female nurses (89%) aged 25 to 34 years (29.8%), working full‐time in LTC homes (53.9%) and with 4 to 7 years of experience (24.6%) represented the most frequent survey categorizations. The College of Nurses of Ontario Registration Statistics Report (CNO, 2022) shows that the most frequent age distribution of RPNs in Ontario is between 24–35 years old (32.4% of all RPNs), working full‐time (62.0%) which makes our sample like the currently registered RPNs in terms of age and employment status.

**TABLE 1:** Summary of demographic information for Ontario RPNs working in Long‐Term Care homes who responded to an Online Survey posted April–August 2021 during the COVID‐19 pandemic.

Demographic characteristic	*N*	Percentage of respondents (%)
Age (years)		
25–34	114	29.8
35–44	92	24.1
45–54	85	22.3
66–64	50	13.1
<25	29	7.6
≥65	6	1.6
Marital status		
Married/ Long‐term relationship	248	64.9
Single	83	21.7
Divorced	23	6.0
Separated but legally married	14	3.7
Widowed	1	0.3
Prefer not to say	7	1.8
Gender		
Female	340	89.0
Male	34	8.9
Other	2	0.5
Status in Canada		
Canadian Citizen	352	92.1
Permanent Resident	20	5.2
Temporary Resident	2	0.5
Other	1	0.3
Primary language		
English	343	89.8
Other	48	12.6
French	6	1.6
Ethnicity		
White/Caucasian	262	68.6
Black	28	7.3
Filipino	22	5.8
South Asian	16	4.2
Prefer not to answer	14	3.7
Indigenous	11	2.9
Other	9	2.4
Latin American	9	2.4
Chinese	8	2.1
Southeast Asian	5	1.3
East Asian	2	0.5
Arab	1	0.3
West Asian	1	0.3
Employment status		
Full‐time RPN	206	53.9
Part‐time RPN	107	28.0
Casual RPN	36	9.4
Other	25	6.5
Not working	17	4.5
Working, not as an RPN	5	1.3
Retired	1	0.3
Years of Practice as an RPN		
4–7	94	24.6
1–3	89	23.3
8–12	74	19.4
≥21	52	13.6
13–20	38	9.9
<1	30	7.9
Household income		
≥$75,000	140	36.6
$50,000–74,999	106	27.7
Prefer not to say	65	17.0
$40,000–49,999	35	9.2
$30,000–39,999	13	3.4
$20,000–29,999	9	2.4
<$5000	7	1.8
$5000–9999	1	0.3
Unionization LTC employment status		
Unionized	332	86.9
Nonunionized	40	10.5
Type of LTC facility		
For profit	146	38.2
Non‐for‐profit, public/municipality	144	37.7
Non‐for‐profit, private	80	20.9
LHIN		
Central East	50	13.1
Hamilton Niagara Haldimand Brant	39	10.2
Champlain	29	7.6
Southwest	28	7.3
Erie St. Clair	24	6.3
Toronto Central	24	6.3
Northeast	23	6.0
North Simcoe Muskoka	21	5.5
Southeast	21	5.5
Central	18	4.7
Waterloo Wellington	17	4.5
Northwest	13	3.4
Mississauga Halton	10	2.6
Central West	6	1.6
Hours per week working in LTC		
20–40	217	56.8
≥40	133	34.8
<20	25	6.5
Role/Job Title		
Staff nurse	309	80.9
Other	62	16.2
RAI‐MDS Coordinator	18	4.7
Infection Prevention and Control	14	3.7
Manager	12	3.1
Director of Care	3	0.8
Clinical Resource Nurse	2	0.5
Occupational Health Nurse	2	0.5
Quality Lead	1	0.3
Employment outside of LTC		
No	271	70.9
Yes, another health sector	84	22.0
Yes, another sector, not health care	25	6.5
Current living situation		
With Partner	190	49.7
With Children <18 years of age	121	31.7
With other family members	82	21.5
With Children ≥18 years of age	59	15.4
Alone	34	8.9
With non‐family members	11	2.9
Prefer not to answer	9	2.4
Other	9	2.4

*Note*: In instances where percentages do not sum to 100, not all respondents answered the survey item. Examples of “other” for employment status included things like “Retired during pandemic”, “Quit during pandemic”, “maternity leave” and “went back to school.” Examples of “other for Role/Job Title” included things like “private nurse”, “behaviour support manager” or “BSO” and “foot care nurse”.

Abbreviations: LHIN, Local Health Integrated Network; LTC, long‐term care.

The influence of COVID‐19‐related factors impacting RPNs working in LTC, such as health changes, modifications in workplace duties, locations and responsibilities are presented in Table 2. Scores for the CD‐RISC‐25 and R@W individual and team scales were presented both as Likert‐scale means and as standardized R@W scores as indicated in the Resilience at Work® Manual (see Table 3; McEwen, 2019a, 2019b). On the CD‐RISC‐25, our sample scored <80 on average and therefore cannot be characterized as having high levels of personal resilience (Connor & Davidson, 2003). On the R@W scale responses were average (i.e., 71%) when compared with standardized scores in the McEwen Resilience at Work® Manual (see Table 3). On this scale, more RPNs were able to develop their capacity to manage Living Authentically (i.e., maintain personal values, use personal strengths, and have good emotional awareness and regulation at work), than they were their capacity to Manage Stress (i.e., maintain work life balance, engage in relaxation, and use work and life routines that help manage everyday stressors), Maintain Perspective (i.e., manage negativity, reframe difficulties and setbacks, and focus on solutions at work) or Build Networks (i.e., develop and maintain workplace and personal support networks). Scores for the TR@W scale are presented both as Likert‐scale means and standardized TR@W scores (see Table 4; McEwen, 2019a, 2019b). On this scale, more RPNs were able to develop their capacity to be Connected (i.e., be cooperative and supportive with each other and encourage a sense of belonging), than they were to develop their Self‐Care (i.e., promote and deploy good stress management routines, respond to overload, and support work‐life balance).

**TABLE 2:** Summary of Responses given by Ontario RPNs working in Long‐Term Care homes regarding the effect of the COVID‐19 pandemic on them gathered by an Online Survey posted April–August 2021 during the COVID‐19 pandemic.

	*N*	Percentage of respondents
Has your household income changed because of the COVID‐19 pandemic?		
No change	149	39.6
Decreased	121	32.2
Increased	106	28.2
Have you applied for any Government income supplements?		
No	273	73.0
Yes	101	27.0
Has your living situation changed as a result of the COVID‐19 pandemic?		
No	291	76.2
Yes	82	21.5
Has your workplace location changed as a result of the COVID‐19 pandemic?		
No change	270	70.7
Other	69	18.1
Reassigned in workplace	19	5.0
Hired via agency to work in LTC	17	4.5
Reassigned to another workplace	8	2.1
Workplace interruptions?		
Medical LOA	104	27.2
Change of employer	83	21.7
Other^a^	75	19.6
Terminated	28	7.3
Compassionate LOA	26	6.8
Change of work sector	20	5.2
Have you ever tested positive for COVID‐19?		
No	327	90.7
Yes, mildly ill	18	4.8
Yes, asymptomatic	14	3.7
Yes, moderately ill	12	3.2
Yes, severely ill	5	1.3
Has your workplace declared an outbreak of COVID‐19 while you were employed there?		
Yes	313	83.2
No	63	16.8
Does your workplace have a policy for informing staff about COVID‐19 cases/outbreaks?		
Yes	336	89.4
No	40	10.6

Abbreviations: COVID‐19, Coronavirus Disease of 2019; LOA, leave of absence.

^a^ Examples of other include “became RPN during pandemic”, “changed health care sectors”, “student”, “contract ended”, “fell and broke my arm”, “self‐isolation”. In instances where percentages do not sum to 100, not all respondents answered the survey item.

**TABLE 3:** Group data for RPN scores on CD‐RISC and R@W Scales.

	*N*	Likert‐scale mean (/7)	Likert‐scale SD	Standardized mean (%)	SD (%)	Min‐max (%)
CD‐RISC‐25 Score	321	–	–	71^a^	14.1	23–99
R@W Total	315	4.7	0.69	66	12.0	32–97
R@W Subscales						
Living authentically	326	5.8	0.9	81	16.2	0–100
Interacting cooperatively	330	5.3	1.1	71	23.2	0–100
Finding your calling	329	5.3	1.2	72	20.2	8–100
Building networks	327	4.7	1.6	63^c^	26.7	0–100
Maintaining perspective	325	4.6^b^	1.4	57	15.1	11–100
Staying healthy	326	4.6	1.3	62	23.1	0–100
Managing stress	323	4.4^b^	1.3	58	22.0	0–100

*Note*: CD‐RISC‐25, Connor‐Davidson Resilience Scale Score (Connor & Davidson, 2003); R@W, Resilience at Work Scale (Winwood et al., 2013); Standardized scores are Likert‐scale scores converted according to the Resilience at Work Research Manual (McEwen, 2019); Scores of 0 in the Min‐Max column indicate Likert scores of 0 (strongly disagree) converted to percentages (i.e., at least one respondent indicated they strongly disagreed to items in that subscale).

^a^ A score below the cut score of 80 as per the CD‐RISC‐25 to characterize the possession of resilience based on the collated self‐reported responses.

^b^ Subscales on which our sample scored lower than Likert‐data provided by Carpio et al. (2018).

^c^ Subscale on which our sample scored lower than standardized data provided by Delgado et al. (2021) and is −1.0 to −0.5 SD below the mean on comparison to normative values for Australian workers (McEwen, 2013).

**TABLE 4:** Group data for RPN scores on the TR@W Scale.

	*N*	Likert‐scale mean (/7)	Likert‐scale SD	Standardized mean (%)	SD (%)	Min–max (%)
TR@W Total	306	4.5	1.21	58	20.2	2–100
TR@W subscales						
Connected	319	4.9	1.59	65	26.5	0–100
Resourceful	315	4.8	1.28	63	21.4	0–100
Perseverance	320	4.8	1.35	64	22.5	0–100
Capability	317	4.7	1.31	62	21.9	0–100
Robust	324	4.6	1.22	74	15.0	17–100
Alignment	319	4.5^a^	1.44	59	24.0	0–100
Self‐Care	314	3.7^a^	1.54	44	25.8	0–100

*Note*: TR@W, Team Resilience at Work Scale (McEwen & Boyd, 2018); standardized scores are Likert‐scale scores converted according to the Resilience at Work Research Manual (McEwen, 2019); Scores of 0 in the Min–Max column indicate Likert scores of 0 (strongly disagree) converted to percentages.

^a^ Subscales on which our sample scored lower than other samples provided by McEwen & Boyd Likert‐data (2018).

**FIGURE 1:**
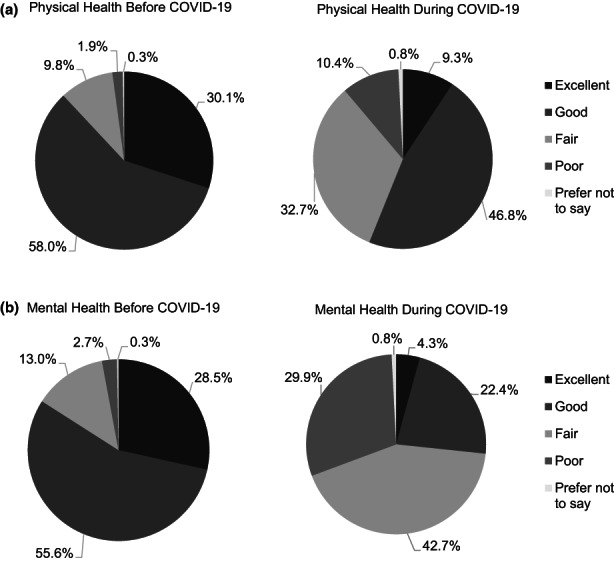
Representation of retrospective self‐reported (a) physical and (b) mental health by RPNs before and during the COVID‐19 pandemic (*N* = 382) working in long‐term care homes collected using an online survey over the months of April–August 2021.

**FIGURE 2:**
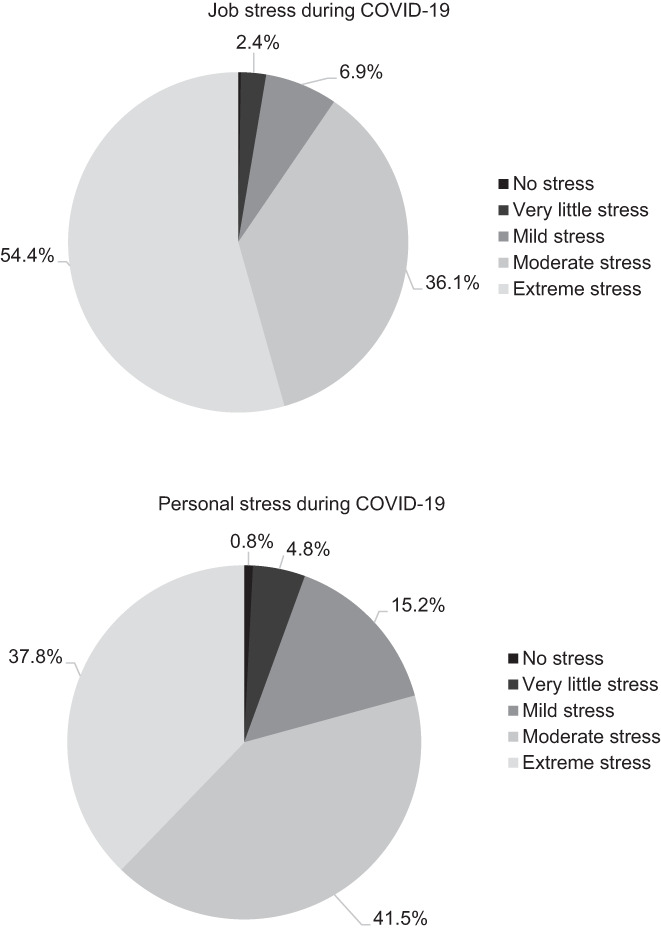
Representation of self‐reported job and personal stress during the COVID‐19 pandemic (*N* = 382) reported by RPNs working in long‐term care homes collected using an online survey over the months of April–August 2021.

Self‐reported current physical and mental health was measured on a 4‐point Likert‐scale. Respondents were also asked to retrospectively rate their physical and mental health on the same scale. The largest changes were in self‐reported physical and mental health were in the categories *excellent* and *fair* (see Figure 1). Respondents reported that their physical and mental health before COVID‐19 was better than during the pandemic (30.1% reported excellent physical health before COVID‐19, and only 9.3% reported excellent physical health during COVID‐19; 28.5% reported excellent mental health before COVID‐19, and only 4.3% reported excellent mental health during COVID‐19). In addition, respondents were asked to rank their personal and job stress while working in LTC during COVID‐19 (see Figure 2). On Likert‐scales, respondents reported extremely high levels of job (54.5%) and personal (37.8%) stress during the COVID‐19 pandemic.

## 5 DISCUSSION

Due to the increasing complexity of health care needs for older adults living in LTC homes, low staffing levels, and the “invisibility” of health care professionals outside of traditional hospital settings (Hewko et al., 2015), supporting resilience of nurses working in the LTC sector of the health care system is critical (Turner, 2014). Our sample scored lower on the *Managing Stress*, *Staying Healthy*, *Maintaining Perspective* and *Building Networks* subscales of the R@W Scale when compared with the *Finding Your Calling*, *Interacting Cooperatively* and *Living Authentically* subscales. The *Maintaining Perspective*, *Managing Stress* and *Building Networks* subscale scores were lower in our sample compared with previous scores in nurses (Carpio et al., 2018; Delgado et al., 2020). On the TR@W Scale, our sample scored lower on the *Self‐Care*, *Alignment*, *Robust*, and *Capability* subscales when compared with the *Perseverance*, *Resourceful*, and *Connected* subscales. These findings indicate that resources and supports for this workforce should focus on things like *Managing Stress*, *Staying Healthy*, *Self‐care*, *and Alignment* rather than things like *Interacting Cooperatively* and *Perseverance*.

The primary findings of this study align with recent studies reporting an exacerbation of the physical and psychological distress experienced by RPNs, with job dissatisfaction and burnout as key contributing factors (LoGiudice & Bartos, 2021; Ou et al., 2021). On the CD‐RISC‐25, our sample scored <80 and therefore cannot be characterized as having capacity for personal resilience. Moreover, for our sample of RPNs, the CD‐RISC‐25 scores are lower than nurses working in intensive care units in New Zealand (73%; Yu et al., 2019), and nurses in Iran responding to the COVID‐19 pandemic (71%; Afshari et al., 2021). However, our sample did score higher on the CD‐RISC‐25 than the America sample of nurses working in LTC (52%; Lin et al., 2021; see Table 3). This aligns with the findings of Baskin and Bartlett (2021) who reported that resilience scores among frontline healthcare workers worldwide were in the moderate range, with nurses in some countries (i.e., The United States; Petzel, 2021) showing a decrease in nurse resilience when compared with pre‐pandemic levels. To our knowledge, for comparison, Canadian nurses' CD‐RISC scores have not been reported previously in the literature.

On the R@W scale, responses were average when compared with data available for mental health nurses (70%; Delgado et al., 2020). In contrast to data presented for nurse managers by Carpio et al. (2018), our sample scored higher on all subscales of the R@W except Finding Your Calling (i.e., having a sense of belonging and purpose at work that fits with the person's core values and beliefs), and the total R@W scale mean score of 4.7 was higher than the 4.2 mean scale score previously reported (Carpio et al., 2018). Our sample also scored lower than comparable samples on the subscales of Maintaining Perspective and Managing Stress. This suggests that the nurses LTC organizations have not been able to successfully develop the infrastructure to allow nurses to have capacity to reframe setbacks, maintain a solution‐focus, and manage negativity and employ work and life routines that help manage everyday stressors while maintaining work‐life balance and ensuring time for relaxation. Due to the data collection methods employed in this research study, we are unable to determine if the RPNs scores reflect deterioration in other components measured by the R@W scale: Living Authentically, Building Networks, Staying Health, Interacting Cooperatively, and Finding Your Calling. The data in this study are reflective of the RPNs working in Ontario LTC currently. It is possible that the data presented in this study are reflective of RPN normative scores, RPN “usual” scores during crisis, or that they are altered by necessity to build their sense of belonging and purpose at work (Finding Your Calling) to compensate for low capacity to reframe setbacks and manage stress (Maintaining Perspective and Managing Stress). On the TR@W scale, our sample scored an average of 4.5/7, which is slightly lower than mean scores reported by McEwen and Boyd (2018). Capacity for Self‐Care and Alignment with their team were low on the TR@W when compared with other samples suggesting that, during this time of crisis, these factors may be more challenging to maintain by RPNs working in LTC and requires assistance with convenience and immediacy.

High levels of resilience contribute to the retention of nurses and helps to sustain their psychological health, by offsetting the personal and professional demands of doing the work of nursing, including the fatigue, burnout, stress, post‐traumatic stress, anxiety, and depression attendant to this work (Yu et al., 2019). Frontline nurses working during the COVID‐19 pandemic experienced stigmatization and the fear of infecting their family members, colleagues (Lorente et al., 2020) and older adult care recipients. Additionally, nurses had to adapt to irreversible and continually fluctuating changes in health and safety care practices, such as infection prevention and control (IPAC) measures, use of personal protective equipment (PPE) mandates, fear of PPE shortages, lack of training and education of infection control practices, and a rapid shift to increased technology use in healthcare, such as videoconferencing, to complete their work (Barrett & Heale, 2021).

The purpose of this study was to explore how RPNs working in LTC during the COVID‐19 pandemic scored on personal and professional resilience assessment measures, and to identify subscales of resilience that resources and supports need to focus on to build a more resilient RPN workforce. A review of healthcare worker resilience during the COVID‐19 pandemic (Baskin & Bartlett, 2021) suggests that building resilience in nurses and other healthcare workers can serve as a protective factor against negative outcomes related to the job, including burnout, anxiety, and depression, and can improve patient outcomes. Therefore, identifying and developing supports for the identified unmet needs in sustaining resilience is critical to maintaining and engaging this workforce in LTC (Clark et al., 2020). Our findings indicate that resources and supports for this workforce should focus on things detailed in the R@W and TR@W scales as *Managing Stress*, *Staying Healthy*, *Maintaining Perspective*, *Building Networks*, *Self‐care*, *Alignment, Robust*, and *Capability* rather than things like *Interacting Cooperatively* and *Perseverance*.

### 5.1 Strengths and limitations

To our knowledge, this is the first study describing the individual and team‐based professional resiliency of RPNs working in LTC homes during the COVID‐19 pandemic. The psychometric properties of the measures of resiliency used in this study were established previously with individuals experiencing stressful situations and thereby are therefore considered robust for use in our sample of RPNs working in LTC homes during the COVID‐19 pandemic. Further, the internal validity, specifically, of the measures used are not currently known. The assessment of individual personal and team‐based professional resilience provided great insight into the intersection of personal and professional resilience in the work‐life of RPNs. We acknowledge the bias inherent with self‐report measures is a limitation of our findings (e.g., recall bias and confirmation bias); however, our data accurately reflect the experiences of the RPNs in our sample. Moreover, our sample was self‐selected (i.e., we are unable to determine why or how participants chose whether to complete our survey). It is possible that nurses who perceived themselves to be more stressed were more likely to respond to the invitation to participate because they wanted to share their experience, or that nurses who perceived themselves to be more stressed were less likely to respond to the invitation to participate because they were at maximum capacity already. We acknowledge that our data may not be generalizable to RPNs in all health care sectors.

### 5.2 Implications for practice

The findings of this study provide evidence to suggest that COVID‐19 resulted in depleted resources among the RPN workforce. This is noteworthy for decision‐makers in professional and workplace organizations to better understand how to use and allocate resources and engage these professionals to retain and rebuild this essential workforce during COVID‐19 and beyond. This study has implications for this shared responsibility between RPNs, their professional association, regulatory bodies, education, and employers. While some elements of resilience depend on the individual, others do not. Based on the results of this study, implications for four stakeholder groups have been identified: (1) the Professional Association WeRPN; (2) The College of Nurses of Ontario; (3) Educators and Curriculum Development Teams; and (4) Employers. First, the WeRPN has developed a Resilience Initiatives program focused on enhancing the self‐care eLearning modules and leadership development courses. A guidebook, *Organizational Resilience: A Guide for Long‐term Care Home to Support Recruitment and Retention of Registered Practical Nurses*, for LTC homes focusing on organizational resilience that supports the RPNs, administrators and managers has been developed (WeRPN, 2022). The guide focuses on how to implement systems and processes to better support RPNs in the workplace and was written in conjunction with the authors of this manuscript. Second, the College of Nurses of Ontario working with Educators and Curriculum Development Teams, have a role to develop curriculum in the identified subscale areas to proactively prepare RPNs for work in LTC homes. The College's Entry to Practice Competencies guide Practical Nursing Curriculum. It is an expectation of all nursing programs to show that the 79 competencies required by the College have been taught, practiced, and assessed during the program. None of the current competencies specifically addresses resilience, despite the critical role resilience plays in this workforce. Teachable constructs of resilience in the form of practice‐based labs in educational programs would benefit RPNs. Finally, moving forward in the pandemic and after, this workforce will continue to see impacts of COVID‐19. Further research is needed to explore how employers can meet the needs of the RPN workforce to address the factors influencing their low subscale scores. The likelihood of future strain arising from changing societal expectations, increased demand for services, and the need for specialized geriatric knowledge with the expanding aging demographic (United Nations, 2015; Statistics Canada, 2014), necessitates the development of evidence‐informed strategies to address these ever‐increasing demands. Our understanding of the existing state of resilience for RPNs in LTC homes will support the development of practice resources, recommendations for practice guidelines, inform institutional and governmental action plans, and influence policy change. Understanding RPNs' experiences during the COVID‐19 crisis is critical to inform the development of social and institutional policy. Current policies, while designed to promote better outcomes for older adults and their families, do not adequately address the complexities of care delivery in LTC homes during a pandemic

## 6 CONCLUSION

Erosion of resilience for individual RPNs working in LTC homes arising from the COVID‐19 pandemic was evident in this study. Resources to support personal self‐care and work‐life balance are needed, and organizational supports to build capacity for team‐based care practices, collegial support in problem‐solving and opportunity for “connecting” with LTC nursing colleagues. Findings suggest a role for personal self‐care, professional development, and workplace solutions for rebuilding this critical workforce to continue caring for older adults living in LTC homes as vulnerable members of our society.

## FUNDING INFORMATION

This research was funded by a SSHRC Partnership Engagement Grant.

## CONFLICT OF INTEREST STATEMENT

The authors have no conflicts of interest to declare.

## ETHICS STATEMENT

Research Ethics Committee approval for the study was obtained from the Western University, Institutional Review Board in London, Ontario.

## Data Availability

The data that support the findings of this study are available from the corresponding author upon reasonable request.

## APPENDIX 1

### Checklist for Reporting Results of Internet E‐Surveys (CHERRIES)

**Table:**

Checklist item	Explanation	Page number
Describe survey design	Describe target population, sample frame. Is the sample a convenience sample? (In “open” surveys this is most likely.)	7
IRB approval	Mention whether the study has been approved by an IRB.	7
Informed consent	Describe the informed consent process. Where were the participants told the length of time of the survey, which data were stored and where and for how long, who the investigator was, and the purpose of the study?	11
Data protection	If any personal information was collected or stored, describe what mechanisms were used to protect unauthorized access.	11
Development and testing	State how the survey was developed, including whether the usability and technical functionality of the electronic questionnaire had been tested before fielding the questionnaire.	7
Open survey versus closed survey	An “open survey” is a survey open for each visitor of a site, while a closed survey is only open to a sample which the investigator knows (password‐protected survey).	7
Contact mode	Indicate whether or not the initial contact with the potential participants was made on the Internet. (Investigators may also send out questionnaires by mail and allow for Web‐based data entry.)	11
Advertising the survey	How/where was the survey announced or advertised? Some examples are offline media (newspapers), or online (mailing lists – If yes, which ones?) or banner ads (Where were these banner ads posted and what did they look like?). It is important to know the wording of the announcement as it will heavily influence who chooses to participate. Ideally the survey announcement should be published as an appendix.	11
Web/E‐mail	State the type of e‐survey (eg, one posted on a Web site, or one sent out through e‐mail). If it is an e‐mail survey, were the responses entered manually into a database, or was there an automatic method for capturing responses?	11
Context	Describe the Web site (for mailing list/newsgroup) in which the survey was posted. What is the Web site about, who is visiting it, what are visitors normally looking for? Discuss to what degree the content of the Web site could pre‐select the sample or influence the results. For example, a survey about vaccination on a anti‐immunization Web site will have different results from a Web survey conducted on a government Web site	11
Mandatory/voluntary	Was it a mandatory survey to be filled in by every visitor who wanted to enter the Web site, or was it a voluntary survey?	11
Incentives	Were any incentives offered (eg, monetary, prizes, or non‐monetary incentives such as an offer to provide the survey results)?	7
Time/Date	In what timeframe were the data collected?	7
Randomization of items or questionnaires	To prevent biases items can be randomized or alternated.	11
Adaptive questioning	Use adaptive questioning (certain items, or only conditionally displayed based on responses to other items) to reduce number and complexity of the questions.	8
Number of Items	What was the number of questionnaire items per page? The number of items is an important factor for the completion rate.	8
Number of screens (pages)	Over how many pages was the questionnaire distributed? The number of items is an important factor for the completion rate.	8
Completeness check	It is technically possible to do consistency or completeness checks before the questionnaire is submitted. Was this done, and if “yes”, how (usually JAVAScript)? An alternative is to check for completeness after the questionnaire has been submitted (and highlight mandatory items). If this has been done, it should be reported. All items should provide a non‐response option such as “not applicable” or “rather not say”, and selection of one response option should be enforced.	8
Review step	State whether respondents were able to review and change their answers (eg, through a Back button or a Review step which displays a summary of the responses and asks the respondents if they are correct).	8
Unique site visitor	If you provide view rates or participation rates, you need to define how you determined a unique visitor. There are different techniques available, based on IP addresses or cookies or both.	11
View rate (Ratio of unique survey visitors/unique site visitors)	Requires counting unique visitors to the first page of the survey, divided by the number of unique site visitors (not page views!). It is not unusual to have view rates of less than 0.1% if the survey is voluntary.	12
Participation rate (Ratio of unique visitors who agreed to participate/unique first survey page visitors)	Count the unique number of people who filled in the first survey page (or agreed to participate, for example by checking a checkbox), divided by visitors who visit the first page of the survey (or the informed consents page, if present). This can also be called “recruitment” rate.	12
Completion rate (Ratio of users who finished the survey/users who agreed to participate)	The number of people submitting the last questionnaire page, divided by the number of people who agreed to participate (or submitted the first survey page). This is only relevant if there is a separate “informed consent” page or if the survey goes over several pages. This is a measure for attrition. Note that “completion” can involve leaving questionnaire items blank. This is not a measure for how completely questionnaires were filled in. (If you need a measure for this, use the word “completeness rate”.)	12
Cookies used	Indicate whether cookies were used to assign a unique user identifier to each client computer. If so, mention the page on which the cookie was set and read, and how long the cookie was valid. Were duplicate entries avoided by preventing users access to the survey twice; or were duplicate database entries having the same user ID eliminated before analysis? In the latter case, which entries were kept for analysis (eg, the first entry or the most recent)?	11
IP check	Indicate whether the IP address of the client computer was used to identify potential duplicate entries from the same user. If so, mention the period of time for which no two entries from the same IP address were allowed (eg, 24 h). Were duplicate entries avoided by preventing users with the same IP address access to the survey twice; or were duplicate database entries having the same IP address in a given period of time eliminated before analysis? If the latter, which entries were kept for analysis (eg, the first entry or the most recent)?	11
Log file analysis	Indicate whether other techniques to analyse the log file for identification of multiple entries were used. If so, please describe.	11
Registration	In “closed” (non‐open) surveys, users need to login first and it is easier to prevent duplicate entries from the same user. Describe how this was done. For example, was the survey never displayed a second time once the user had filled it in, or was the username stored together with the survey results and later eliminated? If the latter, which entries were kept for analysis (eg, the first entry or the most recent)?	11
Handling of incomplete questionnaires	Were only completed questionnaires analysed? Were questionnaires which terminated early (where, for example, users did not go through all questionnaire pages) also analysed?	12
Questionnaires submitted with an atypical timestamp	Some investigators may measure the time people needed to fill in a questionnaire and exclude questionnaires that were submitted too soon. Specify the timeframe that was used as a cut‐off point, and describe how this point was determined.	8
Statistical correction	Indicate whether any methods such as weighting of items or propensity scores have been used to adjust for the non‐representative sample; if so, please describe the methods.	NA

This checklist has been modified from Eysenbach G. Improving the quality of Web surveys: the Checklist for Reporting Results of Internet E‐Surveys (CHERRIES). *J Med Internet Res*. 2004;6(3):e34 [erratum in *J Med Internet Res*. 2012; 14(1): e8.]. Article available at https://www.jmir.org/2004/3/e34/; erratum available https://www.jmir.org/2012/1/e8/. Copyright ©Gunther Eysenbach. Originally published in the *Journal of Medical Internet Research*, 29.9.2004 and 04.01.2012.

This is an open‐access article distributed under the terms of the Creative Commons Attribution Licence (https://creativecommons.org/licenses/by/2.0/), which permits unrestricted use, distribution, and reproduction in any medium, provided the original work, first published in the *Journal of Medical Internet Research*, is properly cited.
